# Editorial: Ecoepigenetics in clonal and inbreeding plants: Transgenerational adaptation and environmental variation, Volume II

**DOI:** 10.3389/fpls.2022.1126610

**Published:** 2023-01-11

**Authors:** Bi-Cheng Dong, Sergio R. Roiloa, Wei Xue, Fei-Hai Yu

**Affiliations:** ^1^ School of Ecology and Nature Conservation, Beijing Forestry University, Beijing, China; ^2^ The Key Laboratory of Ecological Protection in the Yellow River Basin of National Forestry and Grassland Administration, Beijing Forestry University, Beijing, China; ^3^ BioCost Group, Department of Biology, Faculty of Science, University of A Coruña, A Coruña, Spain; ^4^ Institute of Wetland Ecology & Clone Ecology, Taizhou University, Taizhou, China

**Keywords:** clonal growth, environmental change, epigenetics, parental effect, resource provisioning, transgenerational plasticity, inbreeding plants

Accelerating environmental changes at the local, regional, and global scales are likely to favor species that can rapidly adapt to new environmental conditions. Long-lived clonal plants, in which reproduction is mainly asexual and clonal growth plays a central role in their population spread and maintenance, have long been thought to possess low genetic variation so that the potential for genetics-based adaptation to environmental changes may be limited. Thus, epigenetic variation may be particularly important for these clonal plants to adapt to rapid environmental changes (Latzel and Klimešová, 2010; Dodd and Douhovnikoff; Mounger et al., 2021).

Recent work suggests that the performance of an individual (ramet) of clonal plants is influenced by not only its current environmental condition but also the environmental condition of its parents (Huber et al., 2021; Xue et al., 2022). At least three mechanisms can explain such transgenerational (parental or maternal) effects (Herman and Sultan; Luo et al., 2022). First, parental environments could directly influence the performance of clonal offspring by altering the provisioning of carbohydrates and nutrients in vegetative propagules (e.g., fragmented stolons, rhizomes, or storage roots) (Dong et al., 2019). Second, environmental stress could also induce non-provisioning effects between clonal generations, *via* modifying the allocation of defensive chemicals and/or defence-inducing hormones to clonal offspring (Herman and Sultan). Third, parental environments could trigger epigenetic changes in parent plants (e.g., the methylation of DNA and modifications of histones), to facilitate and optimize phenotype variation of clonal offspring in response to environmental change (Douhovnikoff and Dodd, 2015). This Research Topic consists of 11 articles, most of which explore the ecological significance of transgenerational effects and epigenetic variation in clonal plants.

Three papers focus on the relationship between epigenetic regulation and local adaptation of clonal plants under variable environmental stress. By experimental demethylation in natural conditions across different regions of Europe, Sammarco et al. found that the local adaptation mediated by epigenetic variation allowed the stoloniferous plant *Fragaria vesca* to better respond to changing climatic conditions. They suggest that epigenetic-based local adaptation may provide clonal plants with sufficient time to tackle the ongoing environmental crisis and to genetically adapt to it afterwards. In a field experiment, Campoy et al. compared variations in DNA methylation and phenotypic traits between native and introduced populations of the clonal succulent species *Carpobrotus edulis* under a climate change scenario, showing that phenotypic plasticity and global DNA methylation might be related to its rapid adaptation to new habitats. Wang et al. grew experimental populations of the creeping plant *Hydrocotyle vulgaris*, consisting of the same genotype, in two flood regimes and found significant phenotypic differences and associated DNA methylation differentiation between the two types of populations. They suggest that DNA methylation was involved in plant responses to environmental variation.

Four papers consider clonal transgenerational effects on growth, stress tolerance, and competitive ability of clonal offspring. Calibrated with data from two experiments, Wang et al. developed a model to test the transgenerational nitrogen effects on the summed and the mean performance of clonal offspring of the creeping clonal plant *Alternanthera philoxeroides*. They found that transgenerational effects at the whole-generation scale could be jointly influenced by multiple plant inherent characteristics (e.g., the survival rate, the number and the size distribution of clonal propagules), and the magnitude of transgenerational effects could also be obscured by developmental constraints. Zhang et al. tested transgenerational nitrogen effects on the fitness of three generations of the floating clonal plant *Pistia stratiotes*. They found that resource provisioning can increase the initial establishment of clonal offspring in favourable conditions, but this effect may not always be beneficial to their subsequent growth. Yu et al. showed that transgenerational effects could regulate interspecific competition between *P. stratiotes* and *Eichhornia crassipes* by altering the competitive ability of *P. stratiotes*, *via* changes in resource provisioning and/or DNA methylation. Guo et al. tested transgenerational ultraviolet-B (UV-B) effects on the fitness of clonal offspring of the stoloniferous plant *Glechoma longituba*. They found that transgenerational effects could promote the increase in the biomass allocation to aboveground parts in clonal offspring under similar UV-B stress, as well as their defence substances (e.g., flavonoid and anthocyanin), suggesting that the anticipatory transgenerational effects were likely to improve the UV-B resistance.

Two papers examine the effects of population differentiation on the offspring performance of widely-distributed species. Chen et al. examined whether parental environments (i.e., plants were collected from the high and low elevations in the hydro-fluctuation belt of the *Three Gorges Reservoir* region) and the early exposure of offspring of *Polygonum hydropiper* to flooding (accompanied with or without eutrophication) became as a positive or stressful cue on the subsequent growth of these offspring. They found that offspring produced by parental plants in the low elevation might have high adaptability in response to this “predictable” periodic flooding stress. Liu et al. tested the effects of populations with different introduction histories on growth traits of an invasive herb *Erigeron annuus* both in the wild and in common garden experiments. They found that there was parallel genetic and phenotypic differentiation among different invasive populations and that the populations that were introduced earlier had higher genetic diversity and higher growth dominance.

Two papers report within-generation responses of clonal species to stressful environments. Qi et al. tested the interaction effects between arbuscular mycorrhizal fungi (AMF) and soil phosphorus availability on the uptake ability and allocation strategies of an invasive clonal herb *Solidago canadensis*. They found that AMF were able to facilitate phosphorous acquisition by *S. canadensis* in insoluble phosphorous conditions, and also contribute to the invasiveness of *S. canadensis* in the resource-deficient environment. Jing et al. tested the effects of submergence depths on the growth responses of the clonal herb *A. philoxeroides*. They found that *A. philoxeroides* switched from “escape” to “quiescence” strategies in response to increasing submergence depths, and that morphological plasticity such as stem elongation could be essential for the acclimatization of *A. philoxeroides* to water-level fluctuations.

Transgenerational effects in clonal plants have drawn increasing attention during the last few years (Luo et al., 2022). However, there is still a long way to explore in this exciting field. Thus, the knowledge of the mechanisms that relate transgenerational environmental effects to epigenetic inheritance in clonal plants, the correlation between epigenetic variation with genetic and phenotypic variation in wild plant populations, the ecological and evolutionary role of transgenerational effects at different scales (e.g., the individual, population and community levels) have rarely been investigated so far. With the publications on this topic, we hope to further fill in the knowledge gap and stimulate more research on this important issue in the future.

## Author contributions

All authors listed have made substantial and direct intellectual contributions to the work and approved it for publication.
